# The Impact of Neuregulin 4 on Metabolic Dysregulation in Lipodystrophy

**DOI:** 10.1210/endocr/bqaf112

**Published:** 2025-06-28

**Authors:** Leonie Wagner, Juliane Estrada-Kunz, Lisa Roth, Juliane Weiner, Susan Kralisch, Annett Hoffmann, Michael Stumvoll, Mathias Fasshauer, Thomas Ebert, Kerstin Krause, Konstanze Miehle, Anke Tönjes

**Affiliations:** Department of Endocrinology, Nephrology, Rheumatology, University of Leipzig Medical Center, Leipzig 04103, Germany; Department of Endocrinology, Nephrology, Rheumatology, University of Leipzig Medical Center, Leipzig 04103, Germany; Department of Endocrinology, Nephrology, Rheumatology, University of Leipzig Medical Center, Leipzig 04103, Germany; Department of Endocrinology, Nephrology, Rheumatology, University of Leipzig Medical Center, Leipzig 04103, Germany; Department of Endocrinology, Nephrology, Rheumatology, University of Leipzig Medical Center, Leipzig 04103, Germany; Department of Endocrinology, Nephrology, Rheumatology, University of Leipzig Medical Center, Leipzig 04103, Germany; Department of Endocrinology, Nephrology, Rheumatology, University of Leipzig Medical Center, Leipzig 04103, Germany; Department of Nutritional Sciences, University of Giessen, Giessen 35390, Germany; Department of Endocrinology, Nephrology, Rheumatology, University of Leipzig Medical Center, Leipzig 04103, Germany; Department of Endocrinology, Nephrology, Rheumatology, University of Leipzig Medical Center, Leipzig 04103, Germany; German Center for Diabetes Research e.V., Neuherberg 85764, Germany; Department of Endocrinology, Nephrology, Rheumatology, University of Leipzig Medical Center, Leipzig 04103, Germany; Department of Endocrinology, Nephrology, Rheumatology, University of Leipzig Medical Center, Leipzig 04103, Germany

**Keywords:** lipodystrophy, leptin, neuregulin 4, triglycerides

## Abstract

Lipodystrophies (LDs) are rare disorders characterized by the partial or complete loss of subcutaneous adipose tissue, leading to severe metabolic complications. Although metreleptin therapy has shown beneficial effects, its therapeutic efficacy is limited, particularly in patients with partial LD. Neuregulin 4 (NRG4), a batokine secreted by brown adipose tissue, regulates lipid metabolism and hepatic function, but its relevance in LD has not been investigated. In this study, we observed significantly reduced serum NRG4 levels in patients with LD compared to matched healthy controls. NRG4 levels declined further during metreleptin therapy, potentially reflecting fat mass reduction or limited treatment response. To explore functional relevance, we treated a transgenic LD mouse model with recombinant NRG4. While NRG4 enhanced thermogenic gene expression in brown and inguinal white adipose tissue, it did not improve systemic metabolic parameters or hepatic steatosis. In vitro, NRG4 failed to rescue impaired adipogenesis and thermogenesis in brown adipocytes from LD mice but increased insulin-stimulated fatty acid uptake in white adipocytes, indicating a preserved functional response despite differentiation defects. NRG4 also activated hepatic AMPK signaling without improving lipid accumulation. These findings suggest that NRG4 promotes adipose tissue remodeling but is insufficient to restore systemic metabolic homeostasis in LD. Together, our data indicate that NRG4's beneficial effects may depend on the presence of functional adipose tissue, which is profoundly impaired in LD. Consequently, while NRG4 may support local plasticity in adipose tissue, it is insufficient as a therapy for metabolic restoration in LD.

Lipodystrophies (LDs) represent rare inherited or acquired disorders characterized by either a complete or a partial loss of subcutaneous body fat. As a result of insufficient storage capacity of subcutaneous adipose tissue (SAT), triglycerides accumulate in ectopic sites, contributing to metabolic disturbances such as insulin resistance and/or diabetes, dyslipidemia, hypertension, and metabolic dysfunction-associated steatotic liver disease (MASLD) (1). These clinical manifestations closely resemble those seen in patients with obesity, where dysfunctional adipose tissue (AT) is unable to expand further to store excess dietary (2).

In both obesity and LD, impaired AT function disrupts the secretion of various AT-derived factors, known as adipokines. A dysregulation of key adipokines, particularly leptin and adiponectin, has been identified as a major contributor to the metabolic complications observed in both conditions (3, 4). While obesity is typically associated with hyperleptinemia and leptin resistance, LD is marked by leptin deficiency due to the absence of functional AT. Despite their opposing mechanisms, both conditions ultimately lead to metabolic dysfunction, underscoring the importance of balanced leptin signaling for metabolic health (5). Consequently, researchers have prioritized the development of effective treatments for the metabolic complications of both conditions. A landmark study by Oral et al demonstrated that treatment with recombinant leptin (metreleptin) can significantly improve metabolic parameters such as triglyceride levels and glycosylated hemoglobin A1c in patients with LD (6). However, the metabolic outcome of metreleptin treatment is highly variable, with patients with partial LD showing less pronounced metabolic improvements compared to those with generalized LD (7, 8). Given that partial LD represents the most prevalent subtype of LD in Europe (9), a significant proportion of patients suffering from LD do not benefit substantially from metreleptin therapy, highlighting the urgent need to develop alternative treatment strategies.

In recent years, several novel adipokines have been identified that play important roles in interorgan crosstalk to maintain systemic nutrient and energy homeostasis. Among these hormones, neuregulin 4 (NRG4), a batokine secreted by brown adipose tissue (BAT), has emerged as a central regulator of metabolic homeostasis (10). Previous studies have demonstrated that NRG4 plays a primary role in regulating energy expenditure (EE), insulin sensitivity, and glucose metabolism (11). As a ligand, NRG4 predominantly activates the ErbB4 receptor, initiating ErbB3/ErbB4 signaling in hepatocytes and exerting protective effects against diet-induced insulin resistance and hepatic steatosis, partly by attenuating hepatic de novo lipogenesis. Additionally, as a downstream effector of Bmp8b signaling, NRG4 enhances the thermogenic activity in adipocytes by promoting sympathetic axon growth in white adipose tissue (WAT) and BAT (12).

In individuals with partial LD, progressive lipoatrophy of SAT from the extremities, trunk, or gluteal areas is often accompanied by excessive AT accumulation in the facial and cervical regions, which are areas where functionally active BAT has been detected in healthy adults (13). In vivo positron emission tomography/computed tomography analyses of patients with HIV-associated LD or familial partial LD type 2 revealed the absence of BAT activity in dorsocervical SAT following cold stimulation (14, 15). Moreover, histological analysis of cervical AT in these patients revealed an intermediate phenotype, combining features of both BAT and WAT (15). Studies using transgenic aP2-SREBP1c-mice with LDLR-knockout have shown increased sympathetic innervation, uncoupling protein 1 (UCP1) protein expression and *Nrg4* mRNA levels in BAT in response to chronic leptin treatment (16).

The present study aims to elucidate the role of NRG4 in the pathophysiology of LD. To this end, we analyzed a cohort of human patients with LD before and after metreleptin therapy. In addition, we employed a mouse model of LD to investigate the effects of NRG4 on metabolic parameters and primary adipocyte function in LD.

## Materials and Methods

### Characterization of Patients With LD and Healthy Control Subjects

This study included 60 subjects [48 female/12 male, body mass index (BMI) 16.8-33.5 kg/m^2^, age between 16 and 74 years, non-HIV-positive, in detail: familial partial LD: n = 53, acquired partial LD: n = 2, congenital generalized LD: n = 2, acquired generalized LD: n = 3]. We diagnosed LD according to the current multisociety practice guideline based on phenotype and genetic testing whether inherited LD was supposed (17). The control group included 60 metabolically healthy non-LD participants matched for age, sex, and BMI. All subjects were recruited at the Lipodystrophy Centre Leipzig and the outpatient clinic of the University of Leipzig, Department of Endocrinology, Nephrology and Rheumatology, respectively. The study was approved by the local Ethics Committee of Leipzig University (135/13-ek). All subjects gave written informed consent before enrollment in the study.

### Treatment of Patients With LD With Metreleptin

Metabolic criteria for initiation of metreleptin treatment were fulfilled by 16 of the 60 patients with LD (partial LD: n = 14/generalized LD: n = 2; female: n = 13/male: n = 3). For detailed information regarding inclusion and exclusion criteria, as well as metreleptin dosage, we refer to our previous study (18). Recombinant leptin was provided by the manufacturers (Aegerion, Cambridge, MA, USA; Astra Zeneca, London, UK; Bristol Myers Squibb, Munich, Germany; and Amylin, San Diego, CA, USA, respectively) as part of a compassionate use program. Before and at several time points (ie, 1 week, 1 month, 3 months, 6 months, and 12 months) during treatment with metreleptin, all 16 patients received a comprehensive metabolic and anthropometric investigation. Data at 6 and 12 months of metreleptin supplementation were analyzed.

### Laboratory and Anthropometric Analyses of Patients With LD and Controls

Venous blood was taken after a fasting period of at least 8 hours. Metabolic, inflammatory, liver, and renal routine laboratory values were measured at the Institute of Laboratory Medicine, Clinical Chemistry and Molecular Diagnostics of the Leipzig University using standardized laboratory procedures. For the homeostasis model assessment of insulin resistance (HOMA-IR) calculation, the formula provided by Matthews et al (19) was used. The estimated glomerular filtration rate was calculated referring to the CKD-EPI equation by Levey et al (20). We quantified serum concentrations of NRG4 (Phoenix Pharmaceuticals, Burlingame, CA, USA, RRID: AB_3697268), leptin (Mediagnost, Reutlingen, Germany, RRID: AB_2813817), and adiponectin (Mediagnost, RRID: AB_2813736) by means of ELISA, which were applied according to the manufacturer's instructions. The BMI was calculated in the conventional manner [body weight (kilograms) divided by the square of height (meters)], and the waist and hip circumferences were measured to determine the waist-to-hip ratio. For noninvasive assessment of liver steatosis and fibrosis, patients with LD treated with metreleptin underwent conventional abdominal ultrasound and transient elastography (Echosens, Paris, France), with either M- (skin-to-liver-capsule distance <25 mm) or XL-probes (skin-to-liver-capsule distance ≥25 mm) according to the manufacturers’ recommendations before and after 12 months of metreleptin treatment.

### Animal Experiments

The animal experiments were conducted in accordance with guidelines approved by the local authorities of the State of Saxony, Germany, as advised by the local animal ethics review board (TVV39/21). Briefly, transgenic (aP2-SREBP1c) mice, on a C57Bl/6 background, were used as a model for congenital generalized LD (21, 22). Female transgenic (Tg) aP2-SREBP1c mice were treated either with NRG4 or saline (control). Recombinant NRG4 (AdipoGen, San Diego, CA, USA) was administered daily (100 ng/g body weight, IP), for a duration of 20 days. Mice were sacrificed at an age of 19 weeks, and serum and organs were collected and snap frozen.

### Metabolic Phenotyping of Mice

Thirteen days after starting NRG4 treatment, energy metabolism was analyzed in saline-treated (n = 3) and NRG4-treated LD mice (n = 4) by indirect calorimetry using CaloSys V2.1 metabolic chambers (TSE Systems, Bad Homburg, Germany) as previously described (23). The mean oxygen consumption, carbon dioxide production, food intake, and EE were recorded at 5-minute intervals over a 48-hour period. The analysis of indirect calorimetry data was performed using CalR (24).

On day 19 of NRG4 treatment, an intraperitoneal glucose tolerance test was performed. After fasting for 6 hours, mice were injected with 20% glucose solution (10 µL/g body weight). Glucose levels were measured in tail blood taken at 0, 15, 30, 60, 90, and 120 minutes after glucose injection.

### Serum Analysis

Serum levels of murine triglyceride and cholesterol were quantified using the LabAssay Cholesterol (#635-50981, FUJIFILM Wako Pure Chemical Corporation, Tokyo, Japan) and LabAssay Triglyceride (#632-50991, FUJIFILM Wako Pure Chemical Corporation) according to the manufacturer's instructions. Serum levels of circulating leptin and insulin were measured by using ELISA kits (Crystal Chem Cat# 90030, RRID: AB_2722664; and Mercodia #10-1247-01, RRID: AB_2783837, respectively) according to the manufacturer's instructions.

### Histological Staining and Immunohistochemistry

AT samples [inguinal white adipose tissue (iWAT) and BAT] were fixed in 4% formaldehyde at 4 °C for 24 hours. After fixation, the samples were embedded in paraffin and were cut into 7 µm sections. The sections were stained with hematoxylin/eosin following standard protocols. For staining of Mac2 and perilipin, paraffin-embedded sections of iWAT (5 μm) were permeabilized with PBS containing 0.3% Triton (3× 5 minutes), then blocked with 1% BSA (with 0.3% Triton) for 1 hour at room temperature. Sections were incubated overnight at 4 °C with primary antibodies diluted in 1% BSA/Triton: rat anti-MAC2 (1:500, Thermo Fisher, RRID: AB_837132) and rabbit anti-perilipin (1:250, Cell Signaling, RRID: AB_10829911). For enhancement of fluorescent signal, biotinylated goat anti-rat (1:500, Thermo Fisher, RRID: AB_228355) was added. Secondary antibodies included goat anti-rabbit Alexa Fluor 647 (1:500, Invitrogen, RRID: AB_2535813) and extrAvidin-C3 (1:500, Sigma). All were applied for 1 hour at room temperature. Nuclei were stained with 1 μg/mL 4',6-diamidino-2-phenylindole in PBS for 20 minutes at room temperature. Background fluorescence was reduced using 0.3% Sudan black at 50 °C for 2 minutes, followed by distilled water washes. Sections were mounted with Mowiol containing 1% 1,4-diazabicyclo[2.2.2]octane. Frozen liver sections (7 µm) were stained with Oil Red O staining solution (Sigma, St. Louis, MO, USA) for 10 minutes according to the manufacturer's instructions. Microscopic images were captured using a fluorescence microscope (BZ-X800, Keyence, Japan). Areas stained with Oil Red O and Mac2 positive signals were assessed and quantified utilizing ImageJ (National Institutes of Health, Bethesda, MD, USA) software.

### Analysis of Hepatic Cholesterol and Triglyceride Content

Hepatic lipids were extracted from frozen liver samples using HB buffer (pH 7.4) [10 mM NaH2PO4, 1 mM EDTA, 1% polyoxyethylene (10) tridecyl ether] at a ratio of 40 mg tissue per 1 mL buffer by homogenization, followed by centrifugation (13 000 rpm, 10 minutes, 4 °C). Subsequently, the resulting supernatant was heated to 70 °C for 5 minutes. After further centrifugation (13 000 rpm, 10 minutes, 4 °C), the supernatant was collected and proteins were quantified by using the BCA Protein Assay Kit (Thermo Fisher Scientific, Waltham, MA, USA). Cholesterol and triglyceride levels were quantified using the LabAssay Cholesterol (#635-50981, FUJIFILM Wako Pure Chemical Corporation) and LabAssay Triglyceride (#632-50991, FUJIFILM Wako Pure Chemical Corporation) according to the manufacturer's instructions.

### Primary Cell Culture

Stromal vascular fraction was isolated from the BAT and iWAT of aP2-SREBP1c mice that were either Tg or wild-type (WT) (control). Tissues from mice were minced, pooled, and digested in HEPES isolation buffer [100 mM HEPES, 123 mM NaCl, 5 mM KCl, 1.3 mM CaCl2, 5 mM glucose, 4% BSA, 1% ZellShield, 0.2% (w/v) Collagenase II, pH 7.2] at 37 °C for 30 minutes. The suspension was filtered through a 100 µm cell strainer, subsequently placed on ice for 15 minutes to separate from mature adipocytes, and filtered through a 40 µm cell strainer. Erythrocytes were lysed with erythrocyte lysis buffer for 5 minutes. Cells were washed with DMEM (Gibco, Germany) and seeded in DMEM supplemented with 10% fetal bovine serum, 25 mg/mL ascorbic acid, and 1% ZellShield (Minerva, Germany). The medium was changed the first day and then every second day. After reaching confluence (day 0), cells were incubated with differentiation medium (culture medium supplemented with 30 nM insulin, 1 mM rosiglitazone, 0.2 mg/mL dexamethasone) for 2 days followed by culture medium with 30 nM insulin for another 2 days. Subsequently, the medium was changed to normal culture medium for 4 more days (until day 8). From day 0 until day 8 of differentiation, cells were treated with 100 ng/mL NRG4 (AdipoGen) or vehicle (saline). All experiments on primary cells were performed using 4 technical replicates from pooled samples from iWAT or BAT derived from WT or Tg mice (n = 3 and n = 6, respectively).

### Fatty Acid Uptake and Lipolysis In Vitro

Stromal-vascular cells were seeded in 96-well assay plates with black walls and clear bottoms and subjected to adipogenic differentiation in the presence or absence of NRG4 (as described previously). Directly before the addition of the loading buffer, the culture media was replaced by DMEM containing 10 mM insulin (Sigma, # 11376497001). The loading buffer, consisting of 20 mM HEPES buffer and 0.2% free fatty acid-free BSA (Sigma-Aldrich, # A8806) in PBS, was combined with the Fatty Acid Uptake Assay Reagent Component A from the QBT Fatty Acid Uptake Assay Kit (Molecular Devices, #R8132) and then added to the cells. Real-time fatty acid uptake was immediately monitored for 30 minutes using a Tecan Spark multimode microplate reader (Tecan). Data are shown in arbitrary units, reflecting unnormalized raw fluorescence values. For statistical analysis, quantification was based on the final measurement values. For lipolysis cells were differentiated in 96-well plates as described earlier. Before measurement, the culture media was changed to phenol-red free DMEM containing 2% free fatty acid-free BSA and 100 nM isoproteronol (Sigma, # I6504). Lipolysis was measured as the release of fatty acids into the media using non-esterified free fatty acids NEFAHR-2 Assay Reagent2 (Wako Chemicals, #436-91995) following the manufacturer's instructions. Lipid accumulation in primary adipocytes was quantified using AdipoRed Assay™ Reagent (Lonza) as previously described (25).

### RNA Isolation and Quantitative PCR

Isolation of total RNA from tissue and primary cells was performed by using the RNeasy Lipid Tissue Mini Kit (Qiagen, Venlo, The Netherlands). Subsequently, the cDNA was synthesized by using random hexamer primers and M-MLV reverse transcriptase by Promega (Fitchburg, WI, USA). Quantitative real-time PCR was performed using the LightCycler-DNA Master SYBR Green I Kit (Roche, Basel, Switzerland) as previously described (26). Primer sequences are summarized in Supplementary Table S1 (27).

### Western Blot Analysis

Proteins from AT, liver, and primary cells were isolated by using RIPA buffer [150 mM NaCl, 10 mM Tris (pH 7.2), 0.1% SDS, 1% Triton X-100, 1% deoxycholate, 5 mM EDTA] supplemented with complete protease inhibitor cocktail (#11697498001, Roche). The isolated proteins were quantified by using the BCA Protein Assay Kit (Thermo Fisher Scientific) according to the manufacturer's instructions. A total amount of 30 µg protein underwent SDS-PAGE and subsequent transfer to nitrocellulose membranes via tank blot. Membranes were blocked for 1 hour with 3% BSA (Roth, Germany) in Tris-buffered saline with Tween 20 buffer [10 mM Tris (pH 7.2), 5M NaCl, 0,05% Tween 20]. Membranes were incubated with the indicated antibodies overnight at 4 °C [β-actin, RRID: AB_476692; ATP citrate lyase (ACLY), RRID: AB_2223744; AKT, RRID: AB_915783; phosho-AKT (Ser473), RRID: AB_2315049; AMPKα, RRID: AB_330331; p-AMPKα (Thr172), RRID: AB_330330; fatty acid synthase (FASN), RRID: AB_2100798; hormone-sensitive lipase (HSL), RRID: AB_2296900; phosphorylated HSL (Ser660), RRID: AB_2893315; tyrosine hydroxylase (TH), RRID: AB_390204; UCP1, RRID: AB_2241462; phospho-p21 activated protein kinase A (PKA) substrate (RRXS*/T*), RRID: AB_331817; OxPhos, RRID: AB_2533835]. Immunoblots were washed with Tris-buffered saline with Tween 20 buffer, incubated with LI-COR secondary antibodies (680RD goat anti-mouse IgG, RRID: AB_10956588; 800CW goat anti-rabbit IgG, RRID: AB_621843) for 1 hour, and fluorescence intensity was measured using the Odyssey XF Imager (LI-COR, USA). All primary and secondary antibodies and antibody dilutions are listed in Supplementary Table S2 (27).

### Quantification and Statistical Analysis

For statistical analysis of human data, we applied SPSS Statistics Version 27.0 (IBM, Armonk, NY, USA). To decipher significant differences between the human LD and the human control group, we used the nonparametric Mann–Whitney U test. Univariate correlations were determined by Spearman's rank correlation test. For identification of independent relationships between NRG4 and other parameters, multivariate linear regression analysis was performed. Prior to this, relevant parameters were tested for normal Gaussian distribution by the Shapiro–Wilk W test and were logarithmically transformed if they did not show normal distribution. All human data are given as a median and interquartile range, whereas mouse data are depicted as mean ± SE. Statistical analyses of mouse data were performed using GraphPad Prism 10 software (GraphPad, USA). Methods of statistical analyses were chosen based on the design of each experiment and are indicated in the figure legends. Adjusted *P* < .05 was considered statistically significant.

## Results

### Serum NRG4 Levels Are Reduced in Patients With LD

Circulating NRG4 levels were assessed in a cohort of patients with LD and age-, BMI-, and sex-matched healthy controls (Table 1). Our data show significantly lower median NRG4 serum levels in the LD cohort compared to control subjects (2.88 µg/L vs 3.62 µg/L, *P* < .001). Since it has been shown that NRG4 regulates lipid metabolism and whole-body insulin sensitivity in humans and mice (11), we performed a correlation analysis with various cardiometabolic parameters. Univariate correlation analysis revealed significant NRG4 levels with high-density lipoprotein (HDL) cholesterol and a negative correlation with triglycerides (Table 2). Multiple linear regression revealed a negative association between NRG4 and LD status. Additionally, there was a negative association between NRG4 levels and female sex. However, we observed no association between NRG4 and HDL cholesterol or triglycerides (Table 2).

**Table 1.** Baseline characteristics of the study population

	Controls	LD	*P*
n	60	60	
NRG4 (µg/L)	3.62 (1.45)	2.88 (0.90)	**<.001**
Age (years)	39 (22)	42 (24)	.591
Sex (male/female)	12/48	12/48	—
BMI (kg/m^2^)	24.6 (4.9)	25.2 (4.6)	.193
WHR	0.81 (0.11)	0.97 (0.11)	**<.001**
SBP (mmHg)	122 (22)	131 (19)	**<.001**
DBP (mmHg)	78 (15)	81 (14)	.183
HbA1c (%)	5.2 (0.6)	6.0 (2.1)	**<.001**
HbA1c (mmol/mol)	33.3 (6.3)	42.4 (23.0)	**<.001**
FG (mmol/L)	5.2 (0.8)	5.6 (3.8)	**.020**
FI (pmol/L)	51.8 (45.8)	114.9 (113.6)	**<.001**
HOMA-IR	1.7 (1.7)	4.9 (5.8)	**<.001**
Cholesterol (mmol/L)	5.36 (1.35)	5.29 (2.05)	.258
HDL cholesterol (mmol/L)	1.54 (0.59)	0.85 (0.52)	**<.001**
LDL cholesterol (mmol/L)	3.56 (1.39)	2.74 (1.76)	**<.001**
TG (mmol/L)	0.98 (0.60)	2.92 (5.82)	**<.001**
FFA (mmol/L)	0.44 (0.21)	0.61 (0.28)	**.002**
Creatinine (µmol/L)	76 (20)	67 (21)	**.011**
eGFR (mL/min/1.73 m^2^)	94.0 (19.0)	100.2 (31.7)	**.043**
CRP (mg/L)	0.7 (1.5)	1.7 (2.5)	**.016**
Adiponectin (mg/L)	9.3 (7.7)	2.7 (3.7)	**<.001**
Leptin (µg/L)	12.0 (13.9)	4.3 (4.7)	**<.001**
ALAT (µkat/L)	0.34 (0.20)	0.49 (0.42)	**<.001**
ASAT (µkat/L)	0.33 (0.08)	0.48 (0.28)	**<.001**
GGT (µkat/L)	0.26 (0.17)	0.65 (0.60)	**<.001**

Values for median (interquartile range) are shown.

Bold values indicates *P* < .05 as assessed by Mann–Whitney U test.

Abbreviations: ALAT, alanine aminotransferase; ASAT, aspartate aminotransferase; BMI, body mass index; CRP, C reactive protein; DBP, diastolic blood pressure; eGFR, estimated glomerular filtration rate; FFA, free fatty acids; FG, fasting glucose; FI, fasting insulin; GGT, gamma-glutamyl transferase; HbA1c, glycosylated hemoglobin A1c; HDL, high-density lipoprotein; HOMA-IR, homeostasis model assessment of insulin resistance; LD, lipodystrophy; LDL, low-density lipoprotein; NRG4, neuregulin 4; SBP, systolic blood pressure; TG, triglycerides; WHR, waist-hip-ratio.

**Table 2.** Univariate correlations and multivariate regression analysis

	Univariate correlations	Multivariate regression analysis	
	r/*P*	β	*P*
Age (years)	−0.105/.255	—	—
Group (LD vs non-LD)	**—**	**−0**.**359**	.**004***^b^*
Female sex	—	**−0**.**227**	.**011***^b^*
BMI (kg/m^2^)	−0.082/.373	—	—
WHR	−0.177/.055	—	—
SBP (mmHg)	−0.018/.849	—	—
DBP (mmHg)	0.110/.231	—	—
HbA1c (%)	−0.067/.479	—	—
HbA1c (mmol/mol)	−0.074/.437	—	—
FG (mmol/L)	−0.065/.478	—	—
FI (pmol/L)	−0.074/.419	—	—
HOMA-IR	−0.080/.382	—	—
Cholesterol (mmol/L)	0.052/.573	—	—
HDL cholesterol (mmol/L)	**0.204/.025** *^a^*	−0.285	.068
LDL cholesterol (mmol/L)	0.094/.308	—	—
TG (mmol/L)	**−0.291/.001** *^a^*	−0.136	.377
FFA (mmol/L)	−0.101/.283	—	—
Creatinine (µmol/L)	0.048/.602	—	—
eGFR (mL/min/1.73 m^2^)	0.018/.841	—	—
CRP (mg/L)	0.061/.509	—	—
Adiponectin (mg/L)	0.045/.625	—	—
Leptin (µg/L)	0.116/.209	—	—
ALAT	0.023/.815	—	—
ASAT	−0.110/.253	—	—
GGT	−0.099/.302	—	—

Univariate correlations with NRG4 in the entire study population and multivariate regression analysis between NRG4 (log-transformed; dependent variable) and group, sex, HDL cholesterol (log-transformed), and TG (log-transformed). Nonnormally distributed variables were logarithmically transformed prior to multivariate testing. r- and *P*-values, as well as standardized ß-coefficients and *P*-values, are given, respectively.

Bold values indicate a significant correlation.

Abbreviations: ALAT, alanine aminotransferase; ASAT, aspartate aminotransferase; BMI, body mass index; CRP, C reactive protein; DBP, diastolic blood pressure; eGFR, estimated glomerular filtration rate; FFA, free fatty acids; FG, fasting glucose; FI, fasting insulin; GGT, gamma-glutamyl transferase; HbA1c, glycosylated hemoglobin A1c; HDL, high-density lipoprotein; HOMA-IR, homeostasis model assessment of insulin resistance; LD, lipodystrophy; LDL, low-density lipoprotein; NRG4, neuregulin 4; SBP, systolic blood pressure; TG, triglycerides; WHR, waist-hip-ratio.

^*a*^Indicates significant correlation as assessed by Spearman's correlation method.

^*b*^Indicates significant correlation in multivariate analysis.

### Metreleptin Reduces Serum NRG4 in LD Patients

To assess whether leptin replacement affects NRG4 levels, we conducted a prospective study in 16 LD patients treated with metreleptin over 12 months. As shown in Table 3, BMI and serum triglyceride levels decreased significantly after 6 months of treatment with metreleptin. Following the completion of a 12-month treatment course (n = 13), we observed a significant decrease of serum cholesterol levels. Conversely, other glucose and lipid metabolism parameters did not show significant changes in comparison to baseline. As an indicator of patients’ treatment adherence, leptin serum concentration significantly increased between baseline and 6 and 12 months of metreleptin supplementation, respectively. Fat mass significantly decreased after 12 months of metreleptin supplementation (Table 3). Importantly, serum NRG4 levels declined significantly after both 6 and 12 months of metreleptin treatment (baseline: 3.04 µg/L; 6 months: 2.59 µg/L, *P* = .004; 12 months: 2.52 µg/L, *P* < .001; Table 3). Conventional ultrasound measurements of hepatic steatosis during the treatment course were available for 10 patients with LD, indicating improved hepatic steatosis in 5 patients with LD after 12 months of metreleptin supplementation. However, we observed no significant alterations in liver elasticity or controlled attenuation parameter during the treatment (Table 3).

**Table 3.** Baseline characteristics and parameters before and after metreleptin treatment

					Baseline characteristics
n					16
Age (years)					42 (18)
Sex (male/female)					3/13

	Before treatment	6 months treatment	*P*	12 months treatment	*P*
NRG4 (µg/L)	3.04 (1.52)	2.59 (0.85)	**.004**	2.52 (0.99)	**<.001**
BMI (kg/m^2^)	27.4 (5.6)	27.3 (7.1)	**.035**	27.2 (8.1)	.114
WHR	0.97 (0.11)	0.96 (0.09)	.331	0.92 (0.13)	.424
Fat mass (kg)	20.8 (12.7)	N/A		19.8 (16.2)	**.002**
SBP (mmHg)	128 (16)	128 (9)	.754	127 (18)	.576
DBP (mmHg)	80 (16)	74 (14)	.510	77 (17)	.336
HbA1c (%)	8.0 (2.2)	7.2 (1.3)	.081	6.7 (1.7)	.099
HbA1c (mmol/mol)	63.9 (23.8)	55.3 (14.5)	.119	49.7 (18.0)	.107
FG (mmol/L)	9.6 (2.9)	7.9 (4.2)	.808	7.9 (3.8)	.273
FI (pmol/L)	144.2 (280.0)	238.1 (374.4)	.542	110.1 (247.4)	.305
HOMA-IR	12.4 (11.0)	11.6 (22.2)	.583	6.6 (13.9)	.273
Cholesterol (mmol/L)	5.85 (4.94)	5.11 (4.32)	.502	3.87 (3.39)	**.027**
HDL cholesterol (mmol/L)	0.62 (0.48)	0.62 (0.42)	.659	0.79 (0.36)	.530
LDL cholesterol (mmol/L)	1.65 (2.09)	1.66 (1.79)	.318	1.85 (1.44)	.834
TG (mmol/L)	8.64 (14.78)	3.97 (6.18)	**.020**	4.62 (9.85)	.455
FFA (mmol/L)	0.70 (0.30)	0.63 (0.42)	.594	0.56 (0.28)	.162
Creatinine (µmol/L)	63 (20)	62 (25)	1.000	63 (20)	.510
eGFR (mL/min/1.73 m^2^)	101.3 (41.1)	109.3 (33.4)	.893	106.4 (18.0)	.677
CRP (mg/L)	3.1 (5.4)	4.5 (4.5)	.094	3.5 (6.6)	.436
Adiponectin (mg/L)	2.2 (1.8)	2.0 (2.1)	.382	1.8 (1.3)	.331
Leptin (µg/L)	5.1 (4.6)	11.8 (20.6)	**.023**	7.41 (16.9)	**.033**
ALAT (µkat/L)	0.54 (0.46)	0.52 (0.33)	.055	0.45 (0.32)	.173
ASAT (µkat/L)	0.57 (0.57)	0.47 (0.52)	.680	0.43 (0.09)	.213
GGT (µkat/L)	1.04 (2.53)	0.91 (0.74)	.194	0.86 (0.69)	.123
CAP (dB/m)	325 (91)	N/A		359 (168)	.297
Liver elasticity (kPa)	6.9 (3.3)	N/A		5.6 (6.3)	.742

Baseline characteristics and parameters before (n = 16) and at 6 and 12 months (n = 13) after initiation of metreleptin treatment in LD patients, respectively. Values for median (interquartile range) or absolute numbers (n) are shown.

Bold values indicate *P* < .05 as assessed by Wilcoxon signed rank test.

Abbreviations: ALAT, alanine aminotransferase; ASAT, aspartate aminotransferase; BMI, body mass index; CAP, controlled attenuation parameter; CRP, C reactive protein; DBP, diastolic blood pressure; eGFR, estimated glomerular filtration rate; FFA, free fatty acids; FG, fasting glucose; FI, fasting insulin; GGT, gamma-glutamyl transferase; HbA1c, glycosylated hemoglobin A1c; HDL, high-density lipoprotein; HOMA-IR, homeostasis model assessment of insulin resistance; LD, lipodystrophy; LDL, low-density lipoprotein; NRG4, neuregulin 4; SBP, systolic blood pressure; TG, triglycerides; WHR, waist-hip-ratio.

### NRG4 Treatment Does Not Improve Metabolic Parameters in LD Mice

**Figure 1.**
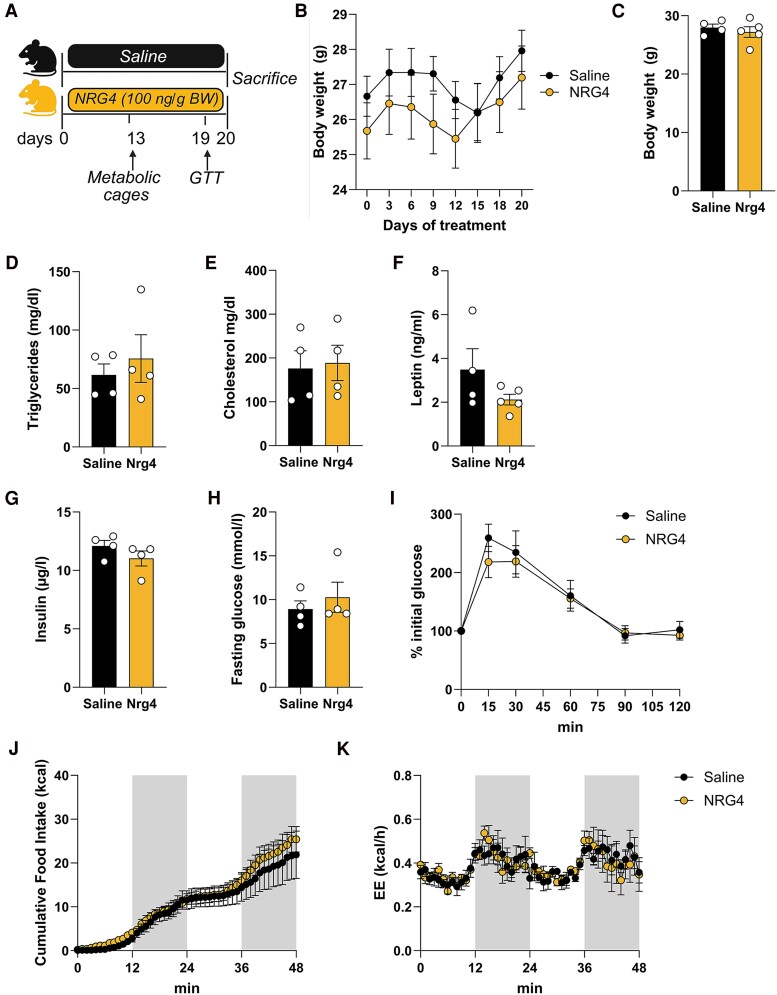
Metabolic phenotyping of NRG4-treated LD mice. (A) Schematic representation of the treatment protocol of female transgenic (aP2-SREBP1c) mice (created with BioRender.com). Tg mice were treated for 20 days with either saline (vehicle) or NRG4 (n = 4 and n = 5 per group, respectively). (B) Body weight development of LD mice during 20-day treatment with saline or NRG4. (C) Body weight and (D-H) serum levels of triglyceride, cholesterol, leptin, insulin, and fasting glucose in saline and NRG4-treated LD mice at the end of the study. (I) Glucose tolerance test at day 19 of NRG4 treatment. (J) cumulative food intake and (K) energy expenditure over 48 hours measured in metabolic cages. All data are presented as means ± SEM. Data in (B), (I), (J), and (K) were analyzed using 2-way ANOVA using time with time and treatment as covariates and Bonferroni post hoc analysis for individual time points. All other data were analyzed using 2-tailed, 2-sided *t*-test. Abbreviations: LD, lipodystrophies; NRG4, neuregulin 4; Tg, transgenic.

To elucidate the potential effects of NRG4 on systemic metabolic homeostasis in LD, female transgenic mice overexpressing aP2-SREBP-1c (Tg) were injected daily with either recombinant NRG4 (100 ng/kg body weight, IP) or vehicle (Fig. 1A). NRG4-treated Tg mice gained slightly more weight, but at the end of the study body weights were not significantly different between the 2 groups (Fig. 1B and 1C). Measurements of plasma metabolites indicated that plasma lipids, including total cholesterol and triglycerides, were comparable between saline- and NRG4-treated LD mice (Fig. 1D and 1E). Serum levels of leptin, insulin, and fasting blood glucose were comparable between groups (Fig. 1F and 1H). Glucose tolerance measured remained unaffected from the 20-day NRG4 treatment (Fig. 1I). Furthermore, indirect calorimetry over 48 hours showed no differences in food intake or EE (Fig. 1J and 1K).

### NRG4 Does Not Activate BAT in LD Mice

**Figure 2.**
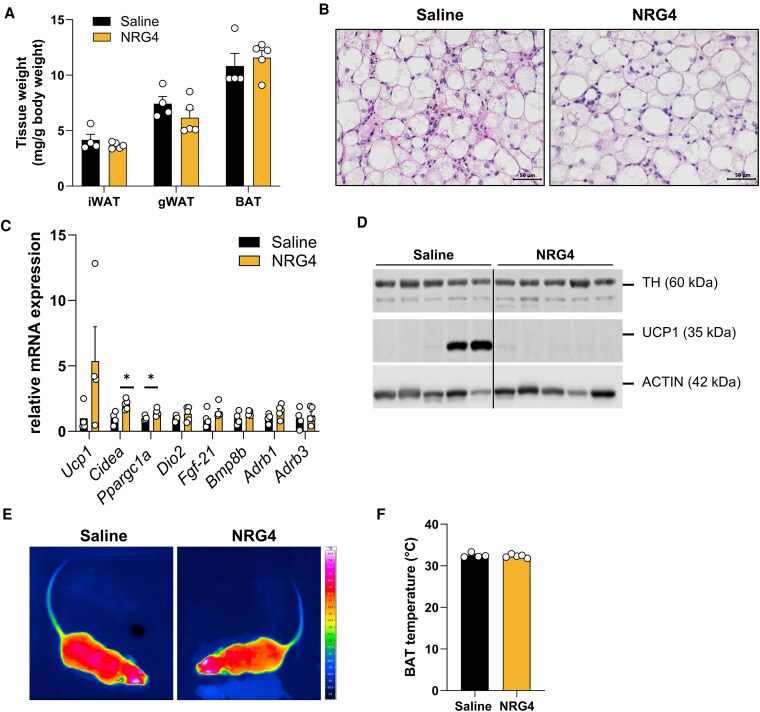
Effects of NRG4 in BAT of LD mice. (A) Tissue weight of iWAT, gWAT, and BAT in Tg mice treated with saline or NRG4 for 20 days. (B) Representative hematoxylin/eosin staining of BAT. Scale bar, 50 µm. (C) Gene expression and (D) protein expression in BAT Tg mice treated with saline or NRG4 for 20 days. (E) Representative infrared pictures and (F) BAT temperatures from Tg mice treated with saline or NRG4 for 20 days. Data represent means ± SEMs. Group size for all data shown: n = 4 mice for saline and n = 5 mice for NRG4-treated group. *P < .05. Data in (A) were analyzed using 2-way ANOVA. Data in (C) were analyzed using 1-way ANOVA, and data in (F) were analyzed using 2-tailed, 2-sided *t*-test. Abbreviations: BAT, brown adipose tissue; gWAT, gonadal white adipose tissue; iWAT, inguinal white adipose tissue; LD, lipodystrophies; NRG4, neuregulin 4; Tg, transgenic.

Since NRG4 has previously described effects on sympathetic innervation and adipose inflammation (12, 28), we first characterized BAT and iWAT in Tg mice treated with either vehicle or NRG4. WAT and BAT weights were comparable between both groups (Fig. 2A). In both experimental groups, BAT displayed a mixed morphology consisting of smaller immature and larger mature adipocytes (Fig. 2B). The larger adipocytes contain large unilocular vacuoles, leading to a visual “whitening” of the BAT. Although thermogenic genes, such as *Cidea* and *Ppargc1a*, were increased in NRG4-treated mice (Fig. 2C), there were no signs of BAT activation. Thus, amounts of UCP1 protein as well as of TH, a marker for sympathetic neurons, were unchanged upon NRG4 treatment (Fig. 2D). Furthermore, the temperature of BAT, the primary site for adaptive thermogenesis in mice, did not change upon NRG4 treatment (Fig. 2E and 2F).

### Cell-autonomous Effects of NRG4 in Brown Adipocytes From Tg Mice and WT Mice

**Figure 3.**
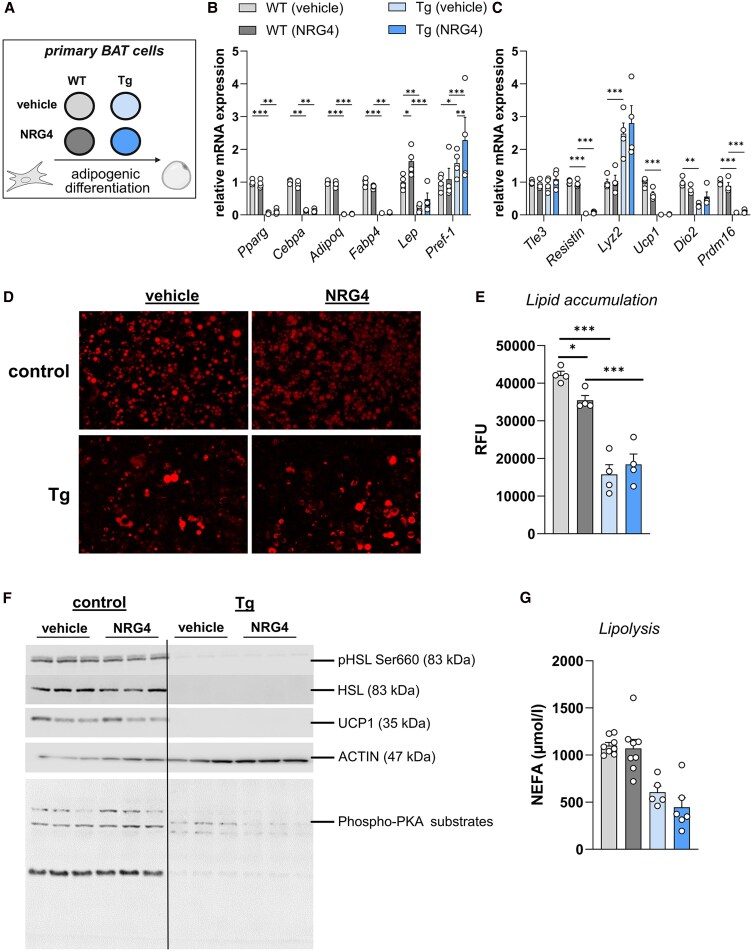
Effects of NRG4 in primary brown adipocytes from WT or Tg mice. (A) Schematic representation of the differentiation protocol of primary brown adipocytes derived from BAT of WT and Tg mice that were treated during adipogenic differentiation with vehicle or 100 ng/mL NRG4 [4 technical replicates from pooled BAT samples of WT or Tg mice (n = 3 and n = 6, respectively)] (created with Biorender.com). (B) Gene expression of adipogenic markers and (C) pan-white adipocyte and thermogenic genes. (D) Representative images of AdipoRed staining indicating accumulation of lipids and (E) quantification of relative fluorescence of stained adipocytes (means of 5 random pictures taken under BZ-X fluorescent microscope). (F) Representative Western blots for phosphorylated HSL, HSL, UCP1, and phospho-PKA substrates in primary adipocytes from BAT of WT or Tg mice that were treated with saline or NRG4 during adipogenic differentiation. Actin was used as loading control. (G) Isoproterenol-stimulated lipolysis in primary adipocytes from BAT of WT or Tg mice treated with either saline or NRG4. Data represent means ± SEM. *P < .05, **P < .01, and ***P < .001. Data in (B) and (C) were analyzed using 2-way ANOVA using time with genotype and treatment as covariates. Data in (E), (F), and (G) were analyzed using 1-way ANOVA. Abbreviations: BAT, brown adipose tissue; HSL, hormone-sensitive lipase; NRG4, neuregulin 4; PKA, p21 activated protein kinase A; Tg, transgenic; UCP1, uncoupling protein 1; WT, wild-type.

We next aimed to investigate the cell-autonomous effects of NRG4 on brown adipocytes. To this end, stromal vascular cells isolated from BAT of Tg mice and littermate WT controls were treated either with NRG4 (100 ng/mL) or vehicle during the entire course of adipogenic differentiation (Fig. 3A). The expression of *Pref-1*, a negative regulator of adipogenesis, was significantly elevated in primary brown adipocytes from Tg mice relative to WT controls and was further elevated by NRG4 treatment (Fig. 3B). Consistent with this, both early and late markers of adipogenic differentiation as well as thermogenic genes were collectively downregulated in brown adipocytes of Tg mice, independent of NRG4 treatment (Fig. 3B and 3C). Additionally, lipid accumulation was markedly reduced in primary brown adipocytes from Tg mice and attenuated by NRG4 in WT controls (Fig. 3D and 3E). Moreover, thermogenic capacity was significantly impaired in primary brown adipocytes from Tg mice, as reflected by reduced phosphorylation of downstream PKA substrates, lack of activating phosphorylation of the HSL, and a significantly lower isoproterenol-stimulated lipolysis (Fig. 3F and 3G). UCP1 protein was detectable exclusively in brown adipocytes derived from WT mice and remained unaffected from NRG4 (Fig. 3F).

### NRG4 Induces Thermogenic Gene Expression in iWAT Without Functional Activation

**Figure 4.**
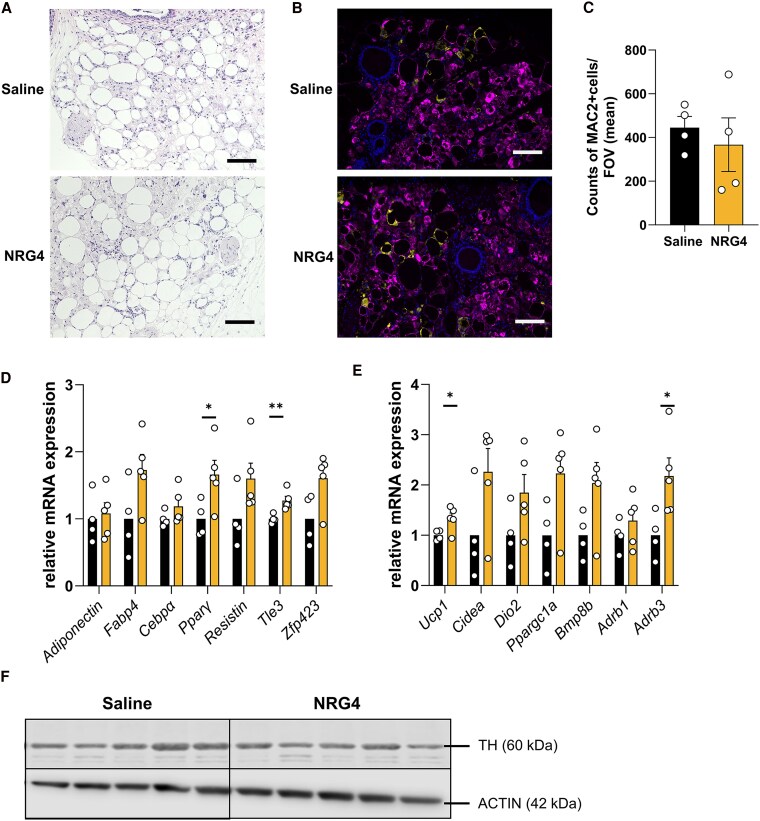
Effects of NRG4 treatment on white adipose tissue in LD mice. (A) Representative hematoxylin/eosin staining (scale bar: 100 µm), (B) MAC-2 staining, and (C) quantification of MAC-2 positive signals per field of visualization of iWAT from Tg mice treated for 20 days with vehicle or NRG4 (scale bar: 100 µm). (D) Gene expression of adipogenic differentiation markers and (E) thermogenic genes in iWAT from Tg mice treated for 20 days with vehicle or NRG4. (F) Representative Western blots for TH in iWAT from Tg mice treated for 20 days with vehicle or NRG4. Actin was used as loading control. Data represent means ± SEMs. Group size for all data shown: n = 4 mice for saline and n = 5 mice for NRG4-treated group. *P < .05. Data in (C) were analyzed using 2-tailed, 2-sided *t*-test. Data in (D) and (E) were analyzed using 1-way ANOVA. Abbreviations: iWAT, inguinal white adipose tissue; LD, lipodystrophies; NRG4, neuregulin 4; Tg, transgenic; TH, tyrosine hydroxylase.

The abnormal morphology of iWAT in Tg mice, characterized by massive inflammation and infiltration of mononuclear cells, was not improved by NRG4 treatment (Fig. 4A). Immunostaining for galectin-3 (MAC-2) showed high levels of MAC-2 protein in crown-like structures within both NRG4-treated and vehicle-treated Tg mice (Fig. 4B and 4C). The expression of common adipocyte marker genes, such as *Fabp4*, *Zfp423, Pparγ,* and its direct target *Tle3,* were significantly upregulated in NRG4-treated LD mice (Fig. 4D). Interestingly, we also observed an increase in expression of thermogenic genes, including *Ucp1* and *Adrb3*, *Cidea*, *Ppargc1a,* and *Dio2*, in iWAT following NRG4 treatment (Fig. 4E). However, protein levels of TH remained unchanged, and UCP1 protein was undetectable in both experimental groups (Fig. 4F).

### Cell-autonomous Effects of NRG4 in White Adipocytes From Tg and WT Mice

**Figure 5.**
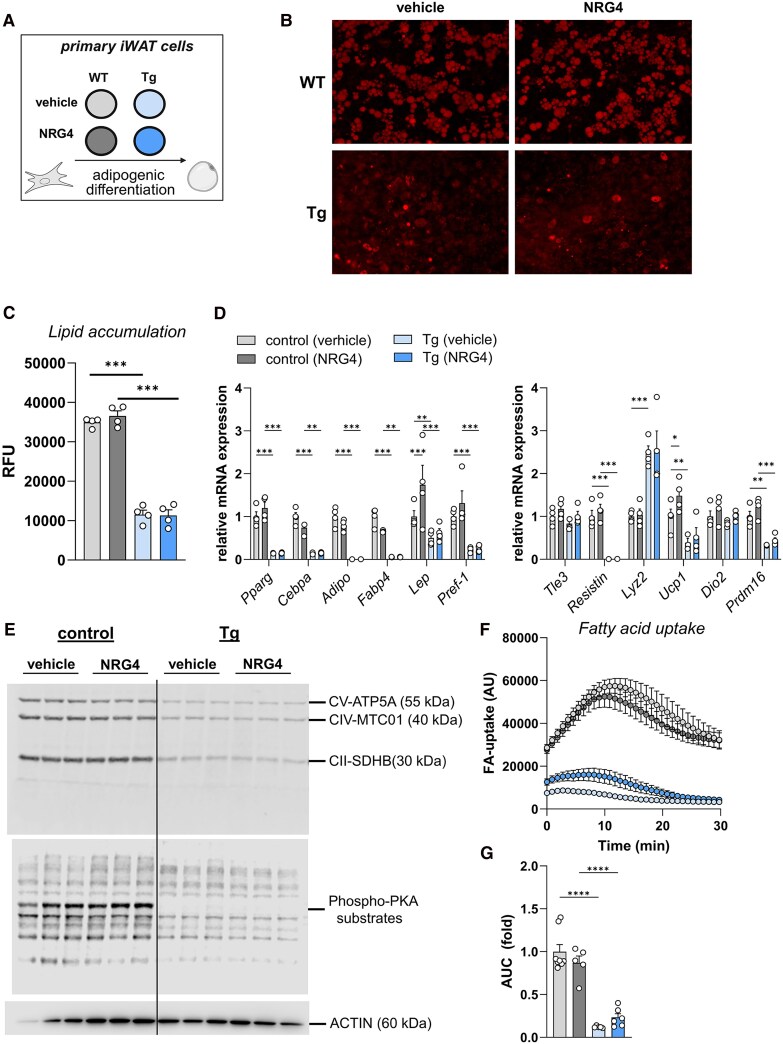
Effects of NRG4 in primary white adipocytes from WT or Tg mice. (A) Schematic representation of the differentiation protocol of primary white adipocytes derived from iWAT of WT and Tg mice that were treated during adipogenic differentiation with vehicle or 100 ng/mL NRG4 [4 technical replicates from pooled iWAT samples of WT or Tg mice (n = 3 and n = 6, respectively)] (created with BioRender.com). (B) Representative images of AdipoRed staining indicating accumulation of lipids and (C) quantification of relative fluorescence of stained adipocytes (means of 5 random pictures taken under BZ-X fluorescent microscope). (D) Gene expression of adipogenic differentiation markers and thermogenic genes and (E) representative Western blots for OxPhos complexes and phospho-PKA substrates in primary adipocytes from iWAT of WT or Tg mice that were treated with saline or NRG4 during adipogenic differentiation. Actin was used as loading control. (F) Kinetic fatty acid uptake in insulin-stimulated primary white adipocytes derived from iWAT of WT or Tg mice that were treated with saline or NRG4 during adipogenic differentiation. (G) Areas under the curve were calculated from fatty acid uptake in (F) and represented as fold change relative to vehicle-treated WT cells. Data represent means ± SEM. *P < .05, **P < .01, and ***P < .001. Data in (C) and (G) were analyzed using 1-way ANOVA. Data in (D) were analyzed using 2-way ANOVA using time with genotype and treatment as covariates. Data in (E) and (F) were analyzed using 1-way ANOVA. Abbreviations: iWAT, inguinal white adipose tissue; NRG4, neuregulin 4; PKA, p21 activated protein kinase A; Tg, transgenic; WT, wild-type.

Primary adipocytes derived from iWAT of Tg and WT mice were treated with either vehicle or NRG4 during adipogenic differentiation (Fig. 5A). Lipid accumulation was reduced in white adipocytes from Tg mice in comparison to those from WT mice, irrespective of NRG4 (Fig. 5B and 5C). There was no effect of NRG4 on markers of adipogenesis and in primary iWAT cells from Tg mice, in accordance with the in vivo findings (Fig. 5D). Contrary to brown primary adipocytes, in white primary cells of Tg mice, *Pref-1* was significantly lower expressed compared to WT mice. Interestingly, NRG4 treatment resulted in a significant increase in *leptin* expression in white primary adipocytes from control WT mice, but not in those from Tg mice (Fig. 5D). This increase in *leptin* was accompanied by significantly higher levels of *Ucp1* mRNA, though no corresponding change was observed at the protein level of UCP1, PKA activation, or mitochondrial OxPhos complex subunits (Fig. 5E). Also, lipolysis induced by isoproterenol was significantly lower in white adipocytes from Tg mice (data not shown). Notably, NRG4 enhanced insulin-stimulated fatty acid uptake in white adipocytes from Tg mice, suggesting a partial functional effect despite impaired adipogenesis (Fig. 5F and 5G).

### Effects of NRG4 Treatment on Liver of LD Mice

**Figure 6.**
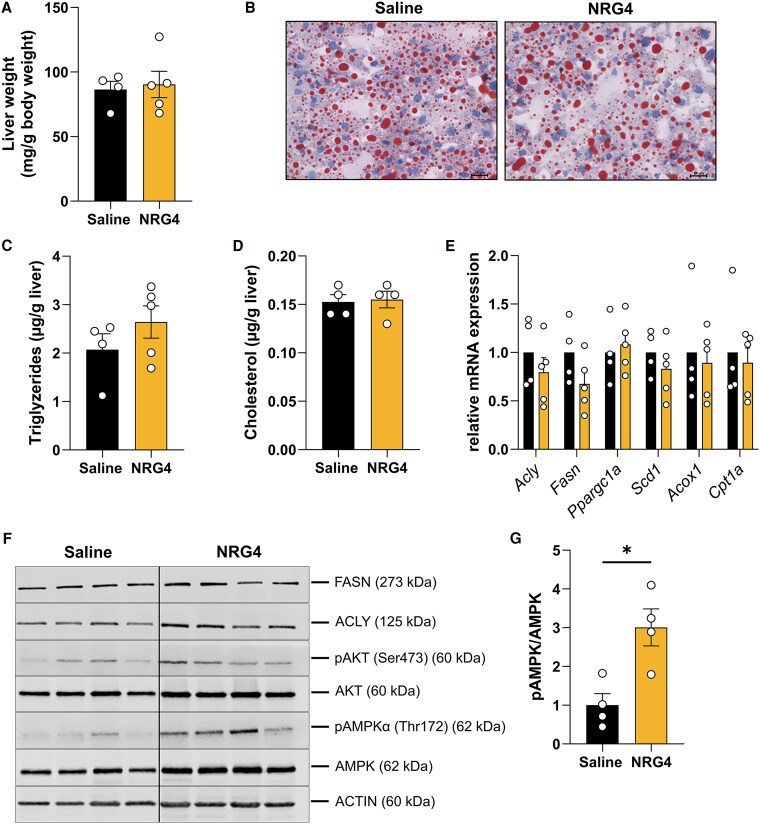
Effects of NRG4 in primary white adipocytes from WT or Tg mice. (A) Liver tissue weight and (B) representative images of accumulation of Oil Red O in frozen liver sections from Tg mice treated with saline or NRG4 for 20 days. (C) Hepatic triglycerides and (D) hepatic cholesterol content in Tg mice treated with saline or NRG4 for 20 days. (E) Gene expression levels of lipogenic genes (*Fasn, Scd1*) and genes involved in fatty acid β oxidation (*Ppargc1a, Scd1, Axox1, Cpt1a*) in the liver of Tg mice treated with saline or NRG4 for 20 days. (F) Representative Western blots for detection of expression of indicated proteins substrates in livers from WT or Tg mice that were treated with saline or NRG4 during adipogenic differentiation. Actin was used as loading control. Data represent means ± SEMs. Group size for are n = 4 mice for saline and n = 4 mice for NRG4-treated group for data shown in (A), (C), (D), (F), and (G). For (E), n = 4 mice for saline and n = 5 mice for the NRG4-treated group were used. Data in (A), (C), (D), and (G) were analyzed using 2-tailed, 2-sided *t*-test. Data in (E) were analyzed using 1-way ANOVA. Abbreviations: NRG4, neuregulin 4; Tg, transgenic; WT, wild-type.

Hepatic steatosis is a common metabolic complication in patients with LD. Previous studies by Wang et al have elucidated that NRG4 reduces hepatic lipogenesis and thereby improves hepatic steatosis (2). No differences in liver weight were observed in NRG4-treated LD mice as compared to saline-treated LD mice (Fig. 6A). NRG4 treatment also did not reduce hepatic lipid accumulation, as shown in Fig. 6B. Furthermore, hepatic triglyceride and cholesterol content was comparable between both experimental groups (Fig. 6C and 6D). The expression levels of key lipogenic genes (such as *Fasn*, *Scd1,* and *Acly*) and genes involved in fatty acid oxidation (such as *Cpt1a*, *Ppargc1a*, and *Acox1*) were not different between NRG4- and saline-treated LD mice (Fig. 6E). Contrary to what was expected, NRG4 administration had no effect on protein expression of fatty acid synthase and ATP citrate lyase (Fig. 6F). Previous studies have established, that NRG4 leads to the activation of autophagy through the AMPK/mammalian target of rapamycin signaling pathway (3). Interestingly, we detected a significant upregulation in the protein level of pAMPKα/AMPKα ratio in NRG4-treated LD mice (Fig. 6F and 6G).

## Discussion

Metreleptin is currently the only specific pharmacological treatment approved for the metabolic consequences of LD. However, a substantial proportion of patients, particularly those with partial LD, show limited clinical response, as reflected by persistently elevated triglycerides, hepatic steatosis, and insulin resistance despite long-term therapy (7, 8). The considerable disease burden and partial therapeutic response underscore the urgent need for alternative or complementary treatment strategies.

NRG4, a BAT-derived batokine, has emerged as a regulator of lipid metabolism, insulin sensitivity, and hepatic lipid handling (11). Despite growing evidence supporting its role in metabolic regulation, data on NRG4 in the context of LD are currently lacking.

In our study, serum NRG4 levels were significantly lower in patients with LD than in matched healthy controls. These findings are consistent with previous reports of reduced circulating NRG4 in individuals with MASLD, metabolic syndrome, gestational diabetes mellitus, chronic kidney disease, and diabetic peripheral neuropathy (29-33). In children with obesity, low serum NRG4 correlates with insulin resistance and MASLD (34), while elevated NRG4 levels are associated with reduced metabolic risk (29). Since LD is also characterized by insulin resistance, chronic low-grade inflammation, and MASLD (17), reduced NRG4 levels in our cohort likely reflect a metabolically adverse phenotype and may be attributable to the loss of functional AT, the primary site of NRG4 production (11).

We further detected a significant positive correlation between NRG4 and HDL cholesterol serum concentrations and a negative association with triglycerides, consistent with findings in newly diagnosed type 2 diabetes (35). However, these associations were not retained in multiple regression analysis, where LD status and female sex emerged as independent negative predictors of circulating NRG4. We did not find a significant association of NRG4 serum levels with parameters of glucose metabolism. This is in contrast to the results of Martinez et al (36), who described a negative correlation of serum NRG4 with insulin sensitivity in a cohort of obese and nonobese participants without diabetes mellitus. However, Martinez and collaborators used the hyperinsulinemic-euglycemic clamp technique in their study, which is the gold standard for assessing insulin sensitivity/insulin resistance in humans. To the contrary, we used the surrogate parameter of HOMA-IR calculation instead of direct measurement of insulin resistance, which has some limitations; eg, HOMA-IR is associated primarily with hepatic insulin resistance and not with peripheral tissue insulin sensitivity (37). Interestingly, serum NRG4 levels further declined during metreleptin treatment. This may be explained by ongoing fat mass reduction, as confirmed in our patient cohort undergoing 12 months’ treatment with metreleptin. Alternatively, low serum NRG4 levels may also reflect the limited metabolic improvements in our patient cohort after 12 months of metreleptin therapy.

To investigate the functional role of NRG4 in LD, we used a Tg mouse model. In contrast to previous studies demonstrating protective effects of NRG4 overexpression or deficiency in high-fat diet models (11), NRG4 treatment in LD mice failed to improve key metabolic parameters, including body weight, plasma lipids, glucose levels, and insulin sensitivity. This discrepancy may be attributed to the near-complete absence of functional AT in LD, which may be essential for NRG4-mediated metabolic benefits.

Nonetheless, NRG4 exerted effects on AT remodeling in LD mice. In BAT, NRG4 treatment led to increased expression of thermogenic genes, such as *Cidea* and *Ppargc1a,* and upregulated *Ucp1* and *Adrb3* in iWAT. However, these changes were not accompanied by increased UCP1 protein expression or energy expenditure, suggesting that transcriptional activation did not translate into functional thermogenesis. In iWAT, NRG4 also promoted expression of adipogenic markers (*Fabp4*, *Zfp423*, *Pparγ*, *Tle3*), suggesting improved differentiation capacity in degenerated WAT. Despite these effects, we did not observe significant changes in TH or canonical markers of axonal outgrowth (*Ntrk2*, *Bdnf,* data not shown), in contrast to previous findings implicating NRG4 in sympathetic remodeling via BMP8B signaling (12, 38).

To further dissect the cell-autonomous effects of NRG4, we performed in vitro differentiation assays in primary adipocytes. In brown adipocytes derived from transgenic LD mice, *Pref-1*, a negative regulator of adipogenesis, was significantly upregulated compared to WT controls and further increased by NRG4 treatment. Both early and late adipogenic markers, as well as thermogenic gene expression, were downregulated, and lipid accumulation was markedly reduced. Functional analyses revealed impaired lipolytic capacity, including reduced PKA substrate phosphorylation, lack of HSL activation, and absent UCP1 protein expression.

In white adipocytes derived from Tg mice, *Pref-1* expression was reduced compared to WT controls, yet both adipogenesis and thermogenic gene expression remained impaired. Treatment with NRG4 did not significantly affect the expression of adipogenic or thermogenic markers in Tg-derived cells, consistent with the in vivo findings. In contrast, NRG4 robustly increased *leptin* and *Ucp1* mRNA expression in white adipocytes from WT controls, although this transcriptional activation was not reflected at the protein level, nor in PKA signaling or mitochondrial OxPhos components. Isoproterenol-stimulated lipolysis was significantly reduced in Tg-derived white adipocytes, further highlighting functional impairment. Notably, NRG4 enhanced insulin-stimulated fatty acid uptake in Tg-derived white adipocytes, indicating a preserved and possibly compensatory functional response despite impaired differentiation.

In the liver, NRG4 treatment had no impact on hepatic steatosis in LD mice, as assessed by liver weight, triglyceride content, and expression of genes involved in lipogenesis and fatty acid oxidation. This contrasts with prior studies in high-fat diet-fed mice, where NRG4 reduced hepatic lipogenesis and improved steatosis (11). It may be likely that the extent of liver dysfunction in the LD model overrides the hepatic effect of NRG4. Nevertheless, we observed a significant increase in hepatic pAMPKα/AMPKα ratios following NRG4 treatment, suggesting activation of the AMPK/mammalian target of rapamycin pathway. As NRG4 has been implicated in modulating autophagy via AMPK signaling (39), this finding raises the possibility that NRG4 influences hepatic stress responses or cellular turnover independent of its effects on lipid storage.

In conclusion, our study identifies significantly reduced serum NRG4 levels in patients with LD, likely reflecting AT loss and associated metabolic dysfunction. In a transgenic LD mouse model, NRG4 treatment enhanced thermogenic gene expression but did not translate into improved metabolic parameters or reduced hepatic lipid accumulation. Together, our data indicate that NRG4's beneficial effects may depend on the presence of functional AT, which is profoundly impaired in lipodystrophy. Consequently, while NRG4 may support local plasticity in AT, it is insufficient as a therapy for metabolic restoration in LD.

## Data Availability

Data sharing is not applicable to this article as no datasets were generated or analyzed during the current study.
