# Correction to: “The Impact of Neuregulin 4 on Metabolic Dysregulation in Lipodystrophy”

**DOI:** 10.1210/endocr/bqaf159

**Published:** 2025-11-14

In above-named article by Wagner L, Estrada-Kunz J, Roth L, Weiner J, Kralisch S, Hoffmann A, Stumvoll M, Fasshauer M, Ebert T, Krause K, Miehle K, and Tönjes A (*Endocrinology*, 2005, 166(9); doi: 10.1210/endocr/bqaf112) there were errors in the authorship roster.

In the original article, in the authorship roster, Konstanze Miehle and Anke Tönjes were only listed as last authors. In the corrected article, Konstanze Miehle and Anke Tönjes are acknowledged as contributing equally to the article with the authors Leonie Wagner and Juliane Estrada-Kunz.

The publisher acknowledges this error.

The article has been corrected online.

doi: 10.1210/endocr/bqaf112

